# A Novel Marker of Inflammation: Azurocidin in Patients with ST Segment Elevation Myocardial Infarction

**DOI:** 10.3390/ijms19123797

**Published:** 2018-11-29

**Authors:** Emrah Ipek, Mustafa Yolcu, Erkan Yildirim, Konca Altinkaynak, Saime Ozbek Sebin, Kamuran Kalkan, Oktay Gulcu, Emrah Ermis, Mustafa Ozturk

**Affiliations:** 1 Department of Cardiology, Istanbul Cerrahi Hospital, 34365 Istanbul, Turkey; 2 Department of Cardiology, Medicana International Hospital, Yeniyuzyil University, 34365 Istanbul, Turkey; yolcudoctor@gmail.com; 3 Department of Cardiology, Biruni University, 34365 Istanbul, Turkey; drerkan23@yahoo.com (E.Y.); emr_ermis@hotmail.com (E.E.); 4 Department of Clinical Biochemistry, University of Health Sciences, Erzurum Training and Research Hospital, 25100 Erzurum, Turkey; kaltinkaynak@hotmail.com; 5 Department of Physiology, Ataturk University School of Medicine, 25100 Erzurum, Turkey; laledeniz21@hotmail.com; 6 Department of Cardiology, University of Health Sciences, Erzurum Training and Research Hospital, 25100 Erzurum, Turkey; kalkankamuran@yahoo.com (K.K.); droktaygulcu@gmail.com (O.G.); mozturk81@yahoo.com (M.O.)

**Keywords:** azurocidine, inflammation, ST segment elevation myocardial infarction

## Abstract

(1) To investigate the role of azurocidin, an antimicrobial protein, in patients with ST segment elevation myocardial infarction (STEMI). (2) This single-center prospective observational study included patients with STEMI and healthy age- and sex-matched control subjects. Baseline demographic, clinical and biochemical data were compared between the two groups. Azurocidin levels at baseline were determined using an enzyme-linked immunosorbent assay. Multivariate linear regression analysis with enter method was used to test the association between azurocidin and independent variables, such as the thrombolysis in myocardial infarction (TIMI) score, synergy between percutaneous coronary intervention with TAXUS and cardiac surgery score, global registry of acute coronary events score, Killip class, C-reactive protein (CRP), and creatinine kinase-myocardial band (CK-MB). (3) A total of 76 patients with STEMI and 30 healthy control subjects were enrolled in the study. Mean ± SD azurocidin levels were significantly higher in patients compared with healthy controls (18.07 ± 13.99 versus 10.09 ± 5.29 ng/mL, respectively). In a receiver-operating characteristic curve analysis, an azurocidin cut-off level of >11.46 ng/mL had 74% sensitivity and 58% specificity in predicting myocardial infarction. Azurocidin levels had a positive correlation with TIMI score (*r* = 0.651). In multivariate linear regression analysis, the TIMI score was an independent predictor of the azurocidin level. (4) Azurocidin is an infection marker that may be important in patients with STEMI.

## 1. Introduction

Coronary artery disease (CAD) is one of the major causes of morbidity and mortality throughout the world [1]. As a major determinant of cardiovascular disease, atherosclerosis is the main cause of ST segment elevation myocardial infarction (STEMI) [1,2]. Although atherosclerosis involves complex pathophysiological mechanisms, inflammation is one of the most important factors in the atherosclerotic process [3,4,5]. The inflammatory process in atherosclerosis also plays an important role in the destabilization of the coronary plaque, which may eventually result in erosion and/or rupture [5]. As a result of this process, a thrombus is superimposed on the eroded or ruptured plaques, resulting in myocardial infarction (MI) [5]. Since STEMI is one of the most devastating presentations of the CAD spectrum, early diagnosis and prompt pharmacological or mechanical reperfusion within 12 h of symptom onset is crucial [6]. Primary percutaneous coronary intervention (PCI) or mechanical reperfusion were reported to be superior to pharmacological reperfusion (fibrinolysis) and patients with higher risk were found to benefit more from PCI [6]. Consequently, risk stratification is important to determine which patients are at a higher risk of morbidity and mortality and this helps the physician to be more aggressive in the management of patients with a higher risk [7]. Additionally, the capacity of risk stratification to reliably identify patients with a lower risk for fatal events can help physicians to choose an early discharge of patients [8]. Several scoring systems have been introduced for risk stratification [8,9,10]. The thrombolysis in myocardial infarction (TIMI) score is a practical and validated scoring system to predict 30-day mortality in STEMI patients [8,9,10,11].

Azurocidin or heparin-binding protein (HBP), which is also known as cationic antimicrobial peptide 37, is a 28 kDa antimicrobial protein included in the serprocidin subgroup of chymotrypsin-like proteases [12,13]. It is stored in azurophilic granules and secretory vesicles of the neutrophils [14]. Neutrophilic adhesion to the endothelium during inflammation induces the basal release of azurocidin from these azurophilic granules and secretory vesicles [13,14]. Azurocidin, as a multifunctional protein, is an important molecule in the host response during infection [12]. The biological functions of azurocidin include antimicrobial activity, induction of monocyte recruitment to the site of inflammation and augmentation of macrophage phagocytosis [12,13,14,15,16,17]. Azurocidin also increases endothelial permeability and macromolecular efflux by breaking cellular barriers [18,19]. Neutrophil degranulation and azurocidin release were reported to be triggered by the M protein from *Streptococcus pyogenes* that leads to toxic shock syndrome, which includes leakage of plasma and multi-organ failure [12,15,17]. Several studies have previously reported that azurocidin is a marker of a worse prognosis in sepsis and acute respiratory distress syndrome [15,16,20].

Considering the role of azurocidin in infection and inflammatory reactions [12,13,14,15,16,17] and its ability to predict worse outcomes in sepsis and acute respiratory distress syndrome (ARDS) [15,16,20], this current study aimed to investigate its role in patients with STEMI.

## 2. Results

A total of 106 individuals including 76 consecutive patients with STEMI who fulfilled the inclusion criteria (mean ± SD age, 60.0 ± 13.6 years; 58 (76.3%) males) and 30 healthy controls (mean ± SD age, 53.4 ± 12.3 years; 20 (66.7%) males) were included in the study. The baseline demographic, clinical and laboratory data of the study group are summarized in Table 1. The patients and the healthy individuals were similar in terms of age, body mass index (BMI), sex, smoking status, admission systolic and diastolic blood pressures. Serum glucose level at admission, low-density lipoprotein cholesterol level, white blood cell (WBC) and neutrophil counts, neutrophil-to-lymphocyte ratio (NLR) and CRP levels were significantly higher in the patient group compared with the healthy controls (*p* < 0.05 for all comparisons). Ejection fraction (EF) was significantly lower in the patient group compared with the healthy controls (*p* < 0.001). A total of seven patients with cardiogenic shock and two patients with cardiogenic pulmonary edema with a median EF of 45% (interquartile range 10–62%) were present in the study group.

**Figure 1:**
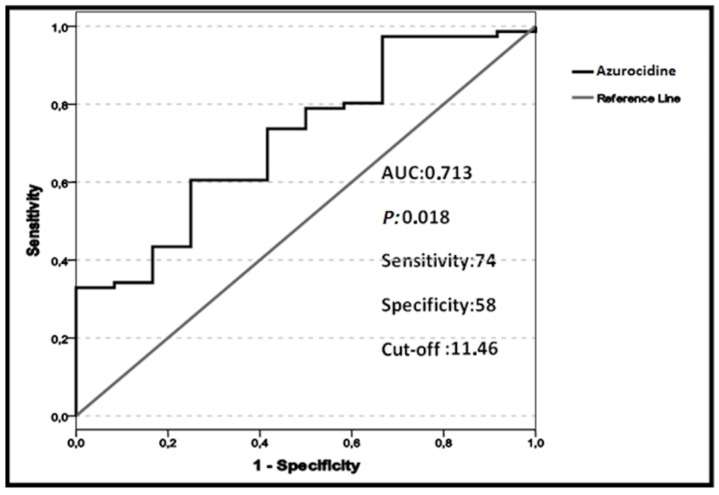
Receiver-operating characteristic (ROC) curve analysis demonstrating the cut-off value of azurocidin for the diagnosis of myocardial infarction (MI) using data from 76 patients with ST segment elevation myocardial infarction and 30 healthy control subjects. The cut-off level of >11.46 ng/mL had 74% sensitivity and 58% specificity in predicting MI (ROC area under the curve (AUC), 0.713; *p* = 0.018).

The mean ± SD azurocidin level was significantly higher in the patient group compared with the healthy controls (*p* = 0.018). To test the power of azurocidin to distinguish patients with MI from healthy individuals, a ROC curve analysis was performed, which demonstrated that an azurocidin cut-off level of >11.46 ng/mL had 74% sensitivity and 58% specificity in predicting MI (ROC area under the curve, 0.713; *p* = 0.018; Figure 1). A total of four patients died during hospitalization because of cardiogenic shock. The azurocidin levels of these patients were found to be greater than the defined cut-off value. 

**Figure 2:**
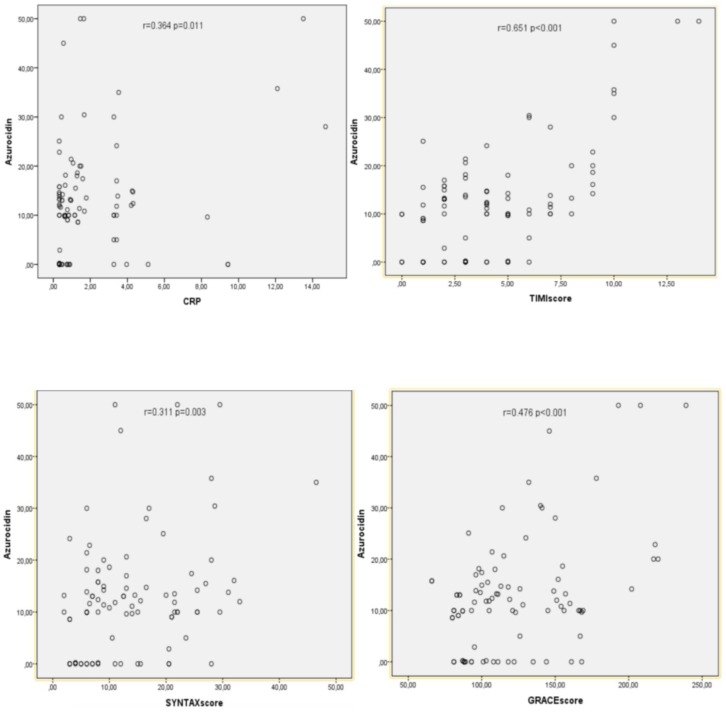
Scatter plots showing the significant correlation between azurocidin levels and C-reactive protein (CRP), thrombolysis in myocardial infarction (TIMI) score, synergy between percutaneous coronary intervention with TAXUS and cardiac surgery (SYNTAX) score and global registry of acute coronary events (GRACE) score. The analysis included 76 patients and correlations were tested with Spearman’s rank correlation coefficient analysis. *r*: correlation coefficient, *p* < 0.01.

The results of the Spearman’s rank correlation coefficient analyses of azurocidin levels with TIMI score, CRP, NLR, CK-MB, SYNTAX, and GRACE scores and Killip class of the patients are presented in Table 2. Azurocidin was found to have a positive correlation with TIMI score (*r* = 0.651, *p* < 0.001). There were also moderate positive correlations between azurocidin levels and Killip class, GRACE score, CRP, and SYNTAX score (*p* < 0.01 for all comparisons). The scatter plots show the correlations between azurocidin levels and TIMI score, CRP, SYNTAX score and GRACE score (Figure 2).

Multivariate linear regression analysis evaluated the association between azurocidin levels and independent variables, including TIMI score, CRP, SYNTAX and GRACE scores and Killip Class, determined by the univariate analysis (*p* < 0.01) (Table 3). There was a positive correlation between azurocidin levels and TIMI score (*p* < 0.001).

## 3. Discussion

The current findings support the fact that inflammation plays a part in the pathophysiological mechanisms involved in STEMI [5]. Two recent animal studies demonstrated the role of inflammation and neutrophils in the atherosclerotic process, healing after myocardial infarction and cardiac remodeling [21,22]. In a previous study, plasma azurocidin levels were found to be significantly higher in patients with acute lung injury (ALI)/ARDS compared with those with cardiogenic pulmonary edema [20]. The authors concluded that azurocidin was a strong prognostic marker for early mortality in ALI/ARDS [20]. However, they could not find any significant difference in azurocidin level between the patients with cardiogenic pulmonary edema and healthy controls [20]. The current study population included a total of seven patients with cardiogenic shock and two patients with cardiogenic pulmonary edema with a median EF of 45% (interquartile range 10–62%) indicating impaired left ventricular function. In contrast to the previous findings [20], the current study may be the first in the literature to demonstrate significantly higher levels of azurocidin in patients with impaired median EF compared with healthy controls. In previous studies, CRP, WBCs, neutrophils and NLR were reported to be related to acute coronary syndromes and can be considered as prognostic markers [23,24]. The patients with STEMI in the current study had significantly higher levels of CRP, WBCs, neutrophils and NLR compared with the healthy control subjects.

The positive correlation between azurocidin levels and the TIMI score of the patients with STEMI in the current study may be consistent with the prognostic role of azurocidin, which has been previously discussed in infection, sepsis and ARDS studies [15,16,19,20]. According to the TIMI risk model defined for STEMI, the 30-day mortality was reported to increase from 0.8% (0 point) to 35.9% (>8 points) and the TIMI risk score was found to have comparable prognostic capability compared with the other risk models [8]. The positive significant correlation of azurocidin levels with the TIMI score in the current study might indicate the sustained inflammatory condition as the disease severity increases. The CRP levels of the patients with STEMI in the current study were significantly higher compared with the control subjects, which was consistent with previous studies [25,26,27]. In one study, higher CRP levels were found to have a prognostic association with in-hospital major adverse cardiac events (MACE) in male patients [25]. The current findings showed that azurocidin levels were positively correlated with CRP levels. This result can guide researchers to design new studies to introduce azurocidin to cardiology practice as a prognostic marker in the future. The significant positive correlation of azurocidin levels with SYNTAX score, GRACE score and Killip class may indicate the association between inflammation and disease severity. However, the relatively lower correlation coefficients indicate lower specificity, which may affect the applicability of these current results.

An emergency service-based study demonstrated that plasma azurocidin level was reported to be well correlated with disease progression to severe sepsis [28]. The authors speculated that a good prognostic biomarker should predict outcomes before the primary outcome becomes apparent and found increased plasma azurocidin levels several hours before the development of circulatory failure or organ dysfunction [28]. The location of azurocidin in the secretory granules, which are the first to be mobilized upon neutrophil activation, can explain this rapid increase [13,18,28]. The mean cut-off value of azurocidin in the emergency service-based multicenter study was set to be 30 ng/mL, which is greater that the cut-off value used in the current study of 11.46 ng/mL [28]. In addition, the median azurocidin concentration was 63.5 ng/mL in organ failure group versus 18.8 ng/mL among patients without organ failure [28]. Interestingly, in their study cohort, 22 out of 85 patients without infection had organ dysfunction with a median plasma azurocidin level of 27.6 ng/mL compared with the 63 non-infected patients without organ dysfunction (11.1 ng/mL) [28]. In the current study, the mean azurocidin level, which was found to be significantly increased in patients with STEMI compared with the control subjects, was 18.07 ng/mL in the patient group. These lower levels of azurocidin in the current study can be explained by the fact that sepsis is a more devastating and mostly irreversible condition and the pathology in a coronary artery segment and its related territory is more localized. In a study of patients with cardiac arrest of mixed origin, the azurocidin levels were found to be an indicator of organ failure and increased among patients with a poor neurological outcome irrespective of the presence of microbiological infection [29]. In a study of intensive care unit (ICU) patients, it was shown that azurocidin levels increased significantly in patients with severe sepsis and septic shock as compared with ICU patients without septic illness [16]. Azurocidin levels at admission were associated with increased risk of death and rising azurocidin levels during hospitalization may help to identify those patients with a deteriorating prognosis [16]. In the current study, four patients died during hospitalization because of cardiogenic shock. Although the number of deaths was insufficient to be able to undertake a mortality analysis, the azurocidin levels of these patients were found to be greater than the defined cut-off value. Although the current study demonstrated that increased plasma azurocidin levels can be predictive of the presence of MI in a group of patients diagnosed as STEMI, because of the relatively lower sensitivity and specificity, an azurocidin measurement alone is insufficient for the detection of MI, but it might be able to be used as a complementary diagnostic marker if validated by future studies. In the acute setting, the selection of patients is performed based on their symptoms and ECG findings. Since the symptom onset of most of the patients is relatively short, the admission troponin and CK-MB levels may not reflect the actual burden of the inflammatory reaction. So, this current study did not compare the sensitivity and specificity of these markers with the levels of azurocidin at admission, which is proposed to be an early prognostic marker. Additionally, this current study was a pilot study and conducted to determine the role of azurocidin in STEMI patients. The aim to select healthy individuals for comparison was to show the increase in azurocidin levels in an extreme inflammatory condition, STEMI, which indicates transmural infarction of the myocardium. A different cut-off level for azurocidin and lower AUC might be observed if patients with unstable angina and NSTEMI had been included in the study.

## 4. Patients and Methods

### 4.1. Study Population

This single-center prospective observational study enrolled consecutive patients admitted to the Emergency Department, Erzurum Training, and Research Hospital, Erzurum, Turkey between October 2015 and January 2016 with the diagnosis of STEMI who were within their first 12 h of symptom onset. The diagnosis of STEMI was made according to the symptoms and ST segment elevation including at least two consecutive derivations on the 12-lead electrocardiogram (ECG) with or without reciprocal ST segment depressions. Blood samples were collected to measure creatinine kinase, creatinine kinase-myocardial band (CK-MB), troponin, C-reactive protein (CRP), complete blood count, serum glucose and lipids, blood urea nitrogen and creatinine tests using an autoanalyzer (ARCHITECT c16000 clinical chemistry analyzer; Abbott Laboratories, Abbott Park, IL, USA) at admission. The total and differential leukocyte counts were measured using an automated hematology analyzer (CELL-DYN Ruby hematology analyzer; Abbott Laboratories). Absolute cell counts were used for analysis. The neutrophil-to-lymphocyte ratio (NLR) was calculated as the ratio of neutrophils to lymphocytes. Inclusion and exclusion criteria are described in full below.

The control group included age- and sex-matched healthy individuals without any conditions defined as the exclusion criteria who were admitted to the outpatient clinic of Erzurum Training and Research Hospital for routine examination. All study participants provided written informed consent. The local ethics committee of Erzurum Training and Research Hospital approved the study in 20 October 2015 (no. 2015/12-110). The study was conducted in accordance with the Declaration of Helsinki.

### 4.2. Measurement of Plasma Azurocidin Levels

In order to measure azurocidin levels, whole blood samples from each patient were collected in a separate tube containing 1.8 mg/mL K_2_-ethylenediaminetetraacetic acid at admission. The blood was then centrifuged for 30 min at room temperature at 1200× *g* using an NF 048 micro and haematocrit centrifuge (NUVE Laboratory and Sterilization Technology, Ankara, Turkey). Plasma samples were stored at −80 °C until further analysis (up to 6 months). Azurocidin levels were measured using an enzyme-linked immunosorbent assay (ELISA) according to the manufacturer’s instructions (E0715Hu, Human Azurocidin (AZU) ELISA Kit; Bioassay Technology Laboratory, Shanghai, China). At 450 nm wavelength with an ELISA reader (PowerWave HT Microplate Spectrophotometer; BioTek, Winooski, VT, USA), the plates were evaluated and there was not any cross-reaction of the assay with any other related protein. The minimum detectable concentration of azurocidin was 0.2 ng/mL. Intra- and interassay coefficients of variation for the ELISA were <8% and <10%, respectively.

### 4.3. Patient Assessments

At admission, a detailed physical examination of all patients was performed and their current smoking status, history of CAD, previous MI, hypertension (HT; systolic blood pressure >140 mm Hg and diastolic blood pressure >90 mm Hg in more than one measurement or receiving antihypertensive drug treatment), diabetes mellitus, and noncardiac diseases such as active or chronic infection, cancer, chronic obstructive pulmonary disease, chronic autoimmune and systemic inflammatory disease, chronic kidney or liver pathology was recorded. Patients with known CAD, prior STEMI, a history of coronary intervention or bypass grafting, known congestive heart failure and/or severe valvular disease, renal failure, autoimmune disease, systemic inflammatory conditions, cancer, hematological disorders, acute or chronic infection of any organ system and any drug therapy that may affect the measurement of azurocidin were excluded. All of the patients were assessed according to the CAD risk factors and Killip classification [30] and all of these data were recorded. The admission TIMI and in-hospital death global registry of acute coronary events (GRACE) risk score points were calculated in order to predict poor in-hospital and post-discharge outcomes. A TIMI score of 0–14 was possible using criteria of age, presence of diabetes/HT or angina, heart rate < 100 beats per min, systolic blood pressure < 100 mmHg, Killip class II–IV, weight < 67 kg, anterior MI or left bundle branch block at presentation, and time to treatment > 4 h [8]. The TIMI risk score was calculated using an online tool [31]. The GRACE risk score points (age, creatinine, heart rate, systolic blood pressure, Killip class, presence of cardiac arrest at admission, cardiac marker elevation, and ST segment changes) were also recorded [32]. The GRACE risk score was calculated using an online tool [33]. The body mass index (BMI) was obtained by division of weight (kg) by the square of the height (m).

Transthoracic echocardiography to measure the left ventricular ejection fraction (EF) and valvular function (GE Vivid™ 7 Ultrasound Machine; GE Healthcare, Piscataway, NJ, USA) was undertaken during the initial evaluations. Greater than 50% stenosis in one of the major coronary arteries was assumed to be significant. The synergy between percutaneous coronary intervention with TAXUS and cardiac surgery (SYNTAX) score of the patients were calculated using an online tool [34] by two invasive cardiologists (Emrah Ipek and Emrah Ermis) in order to determine the severity and complexity of the CAD [35].

### 4.4. Statistical Analyses

All statistical analyses were performed using PASW, version 18.0 (SPSS Inc., Chicago, IL, USA) for Windows^®^. Continuous variables are presented as mean ± SD or median (interquartile range) and categorical variables are presented as *n* of patients (%). The Kolmogorov–Smirnov test was used to test the normality of the distribution of continuous variables. Statistical analysis of data between two groups was performed using unpaired *t*-test for parametric data and Mann–Whitney *U*-test for nonparametric data. Correlations were tested with Spearman’s rank correlation coefficient. χ^2^-test was used for categorical variables. Multivariate linear regression analysis with enter method was used to test the association between azurocidin and independent variables, such as TIMI, SYNTAX and GRACE scores, Killip class and CRP determined by the univariate analyses (*p* < 0.01). The results were given as regression coefficient (β) and 95% confidence intervals (CI). Receiver-operating characteristic (ROC) curve analysis was used to determine the optimum cut-off level of azurocidin that would predict MI. A two-tailed *p*-value < 0.05 was considered statistically significant.

## 5. Study Limitations

This current study had several limitations. The major limitation was the small sample size. The lack of azurocidin measurements at discharge and on the 30th day post-discharge is another limitation. Additionally, the exclusion of patients with known CAD, prior STEMI, a history of coronary intervention or bypass grafting, known congestive heart failure and/or severe valvular disease, renal failure, autoimmune disease, systemic inflammatory conditions, cancer, haematological disorders, acute or chronic infection of any organ system and any drug therapy that may affect the measurement of azurocidin, may have limited the generalizability of the results.

## 6. Conclusions

In conclusion, azurocidin is an infection marker that may be important in patients with STEMI. To date, this current study is the first to evaluate the role of azurocidin in a cohort of patients without an infection. As this was a pilot study, it would not be appropriate to use azurocidin in daily practice to predict the prognosis at admission or to use it in the decision-making process until further large-scale studies have been conducted. However, azurocidin may be used in the future as a complimentary diagnostic and/or prognostic marker if it is evaluated and validated in studies with a larger sample size. Studies investigating patients with other presentations of CAD such as non-STEMI, unstable angina and/or stable angina pectoris, are needed.

## Figures and Tables

**Table 1:** Baseline demographic, clinical and laboratory data of patients (*n* = 76) with ST segment elevation myocardial infarction (STEMI) and age- and sex-matched healthy control subjects (*n* = 30).

Characteristics	Patients with STEMI	Control Subjects	Statistical Significance ^a^
	*n* = 76	*n* = 30	
Age, years	60.0 ± 13.6	53.4 ± 12.3	NS
Sex, males	58 (76.3)	20 (66.7)	NS
BMI	24.08 ± 6.99	20.25 ± 7.94	NS
Smokers	38 (50.0)	17 (56.7)	NS
Hypertension	34 (44.7)	0 (0.0)	NA
SBP, mmHg	126 (50–220)	115 (100–125)	NS
DBP, mmHg	80 (30–88)	75 (50–81)	NS
Diabetes mellitus	59 (77.6)	0 (0.0)	NA
Glucose, mg/dL	128 (79–361)	94 (89–105)	*p* = 0.02
Hyperlipidaemia	65 (85.5)	0 (0.0)	NA
LDL-C, mg/dL	133.37 ± 39.14	92.0 ± 12.2	*p* = 0.042
WBC, 10^3^/µL	11.97 (6.79–22.05)	8.09 (6.79–8.88)	*p* < 0.001
Neutrophil, 10^3^/µL	9.43 ± 3.69	5.78 ± 1.16	*p* < 0.001
Lymphocyte, 10^3^/µL	1.76 (0.6–9.4)	1.65 (0.78–2.56)	NS
NLR	7.97 ± 4.10	4.56 ± 2.61	*p* = 0.034
CRP, mg/dL	1.47 ± 2.80	0.39 ± 1.12	*p* = 0.028
Creatinine, mg/dL	0.87 ± 0.18	0.84 ± 0.17	NS
EF, %	45 (10–62)	65 (60–65)	*p* < 0.001
CK-MB, mg/dL	52.0 ± 12.4	–	NA
Azurocidin, ng/mL	18.07 ± 13.99	10.09 ± 5.29	*p* = 0.018
TIMI score	4.5 ± 2.9	–	NA
SYNTAX score	14.9 ± 9.7	–	NA
GRACE score	122.6 ± 35.0	–	NA

Data expressed as mean ± SD, median (interquartile range) or *n* of patients (%). ^a^ Statistical analysis of data between the two groups was performed using unpaired *t*-test for parametric data, Mann–Whitney *U*-test for nonparametric data and χ^2^-test for categorical variables. BMI, body mass index; SBP, systolic blood pressure; DBP, diastolic blood pressure; LDL-C, low-density lipoprotein cholesterol; WBC, white blood cells; NLR, neutrophil-to-lymphocyte ratio; CRP, C-reactive protein; EF, ejection fraction; CK-MB, creatinine kinase-myocardial band; TIMI, thrombolysis in myocardial infarction; SYNTAX, synergy between percutaneous coronary intervention with TAXUS and cardiac surgery; GRACE, global registry of acute coronary events; NA, not applicable; NS, no significant between-group difference (*p* ≥ 0.05).

**Table 2:** Spearman’s rank correlation coefficient analysis of azurocidin levels with baseline demographic and clinical characteristics of patients (*n* = 76) with ST segment elevation myocardial infarction.

Characteristic	Azurocidin Levels	
	Correlation Coefficient *r*	Statistical Significance ^a^
TIMI score	0.651	*p* < 0.001
CRP	0.364	*p* = 0.011
NLR	0.110	NS
CK-MB	0.104	NS
SYNTAX score	0.311	*p* = 0.003
GRACE score	0.476	*p* < 0.001
Killip class	0.505	*p* < 0.001

^a^ Correlation was significant at the *p* < 0.01 level (2-tailed). TIMI, thrombolysis in myocardial infarction; CRP, C-reactive protein; NLR, neutrophil-to-lymphocyte ratio; CK-MB, creatinine kinase-myocardial band; SYNTAX, synergy between percutaneous coronary intervention with TAXUS and cardiac surgery; GRACE, global registry of acute coronary events; NS, no significant correlation (*p* ≥ 0.01).

**Table 3:** Multivariate linear regression analysis to evaluate the association between azurocidin levels and independent variables identified in the univariate linear regression analysis.

	Dependent Variable: Azurocidin	
Independent Variables	β (95% CI)	Statistical Significance ^a^
TIMI score	0.642 (0.337, 0.947)	*p* < 0.001
GRACE score	0.184 (−0.476, 0.109)	NS
SYNTAX score	0.041 (−0.140, 0.225)	NS
Killip class	0.169 (−0.076, 0.413)	NS
CRP	0.077 (−0.095, 0.248)	NS

^a^ Linear regression analyses using the enter method were used for the multivariate analysis of independent variables that were included if they were significantly different in the univariate analyses (*p* < 0.01). CI, confidence interval; TIMI, thrombolysis in myocardial infarction; GRACE, global registry of acute coronary events; SYNTAX, synergy between percutaneous coronary intervention with TAXUS and cardiac surgery; CRP, C-reactive protein; NLR, neutrophil-to-lymphocyte ratio; NS, no significant correlation (*p* ≥ 0.05).
